# MIRA: an R package for DNA methylation-based inference of regulatory activity

**DOI:** 10.1093/bioinformatics/bty083

**Published:** 2018-03-01

**Authors:** John T Lawson, Eleni M Tomazou, Christoph Bock, Nathan C Sheffield

**Affiliations:** 1 Department of Biomedical Engineering, University of Virginia, Charlottesville, VA, USA; 2 Center for Public Health Genomics, University of Virginia, Charlottesville, VA, USA; 3 Children’s Cancer Research Institute, St. Anna’s Kinderkrebsforschung, Vienna, Austria; 4 CeMM Research Center for Molecular Medicine of the Austrian Academy of Sciences, Vienna, Austria

## Abstract

**Summary:**

DNA methylation contains information about the regulatory state of the cell. MIRA aggregates genome-scale DNA methylation data into a DNA methylation profile for a given region set with shared biological annotation. Using this profile, MIRA infers and scores the collective regulatory activity for the region set. MIRA facilitates regulatory analysis in situations where classical regulatory assays would be difficult and allows public sources of region sets to be leveraged for novel insight into the regulatory state of DNA methylation datasets.

**Availability and implementation:**

http://bioconductor.org/packages/MIRA.

DNA methylation interacts with other regulatory features to control gene expression (Stadler *et al.*, 2011). The connection between methylation and transcription factor (TF) binding goes both ways: TF binding affects and is affected by DNA methylation (Zhu *et al.*, 2016), making it difficult to infer the causative factor; nevertheless, independent of directionality, the inverse correlation between DNA methylation and gene expression indicates that regulatory information can be derived from DNA methylation data.

Multiple approaches have been used to relate DNA methylation to regulatory activity; for example, correlating differential methylation with expression of nearby genes (Yao *et al.*, 2015), or testing enrichment of TFs in differentially methylated regions (Wijetunga *et al.*, 2017; Yao *et al.*, 2015). These approaches are limited by arbitrary thresholds for differential methylation and do not make full use of genome-wide data. Also, factors other than DNA methylation levels, such as the shape of the DNA methylation profile around a site, may be important to the site’s activity (Kapourani and Sanguinetti, 2016).

We recently introduced and validated a novel method called MIRA (Methylation-based Inference of Regulatory Activity), which takes advantage of genome-scale DNA methylation data to assess regulatory activity (Sheffield *et al.*, 2017). We now present the MIRA R package which enhances this method and makes it broadly available.

MIRA requires two inputs: (i) single-nucleotide-resolution DNA methylation data; and (ii) a set of genomic regions (Fig. 1A). The DNA methylation data could come from sources such as whole genome or reduced representation bisulfite sequencing (WGBS or RRBS), or microarrays. MIRA has been successfully tested with coverage as low as 450k array data. Genomic regions can be derived from sequencing assays such as ChIP-seq, DNase-seq, or ATAC-seq. Many region sets are publicly available through large-scale genomics projects and may be conveniently accessed through R packages like LOLA (Sheffield and Bock, 2016).

**Fig. 1.**
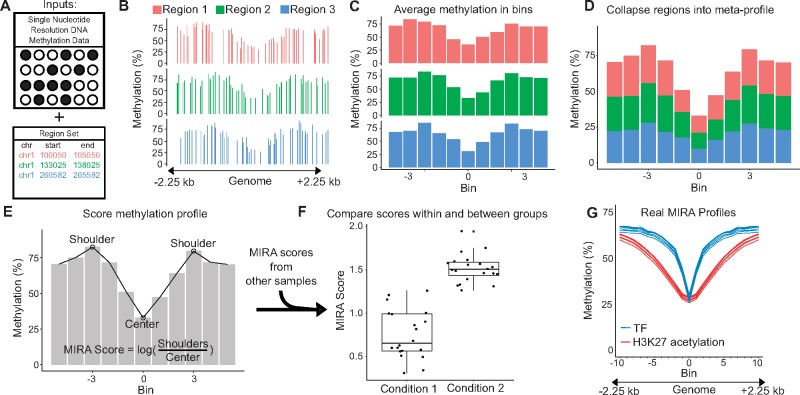
MIRA workflow. (**A**) Two inputs to MIRA: DNA methylation data for the sample of interest and a set of genomic regions that share a biological annotation. (**B**) Three regions from the region set are shown for this example, but a region set would normally be composed of thousands of regions. The DNA methylation level at individual CpGs is plotted for each 4.5 kb region, which is centered around a site of interest. (**C**) Each region is split into 11 bins of approximately equal size and an average methylation level is calculated based on the CpGs in each bin. (**D**) All regions are aggregated into a single DNA methylation profile by averaging methylation from the corresponding bins of each region. (**E**) The methylation profile is scored by taking the log of the ratio between the average methylation of the two shoulders and the methylation of the center. An algorithm determines the position of the shoulders. (**F**) As might be seen in an experiment that uses MIRA, the single score calculated from this sample is compared to scores from other samples of the same type—condition 1—as well as to samples of a different type—condition 2. All scores were calculated using the same region set. The difference in scores between groups suggests differential activity of this region set. (**G**) Real MIRA profiles for a TF region set and for an H3K27 acetylation region set with DNA methylation data from six mesenchymal stem cell samples

Using these two inputs, MIRA aggregates the DNA methylation of individual CpGs to create a summary profile through several steps: First, each region (Fig. 1B) is split into *n* bins. Second, the DNA methylation level (0–100%) within a bin is averaged (Fig. 1C). Third, the regions are aggregated into a single summary profile by averaging the DNA methylation levels of each bin across all regions (Fig. 1D). MIRA thus creates a ‘meta-region profile’ that provides general information about the activity of that region type across the genome. Through aggregation, MIRA handles sparse DNA methylation data well. This makes MIRA well suited for low-coverage bisulfite sequencing (e.g. Farlik *et al.*, 2015).

Once an aggregate profile is constructed (Fig. 1E), it is scored to quantify the regulatory activity (Fig. 1F). MIRA assumes that genomic regions with lower DNA methylation levels have higher regulatory activity and gives a score based on the deepness of the ‘dip’ in the middle of the ‘meta-region profile’. MIRA automatically determines the location of the edges of the dip and calculates the score as the natural logarithm of the ratio between the DNA methylation level of the edges of the dip and the DNA methylation level of the center of the dip (Fig. 1F). The score reduces the DNA methylation profile to a number, which predicts the region set’s aggregate regulatory activity. MIRA scores can be compared between samples to identify regulatory differences.

MIRA supports a variety of applications depending on the context and what type of region set is used. For example, MIRA can be used to compare the chromatin states of different types of cells (Sheffield *et al.*, 2017). MIRA makes analysis of regulatory activity possible in cases where it would otherwise be infeasible. When sample amount or quality would not allow ATAC-seq or ChIP-seq but DNA methylation data can be obtained, regulatory analysis can be done with MIRA using existing ATAC-seq or ChIP-seq data (e.g. from a database). MIRA is also valuable for cases where it would be impractical in terms of time or cost to perform traditional regulatory assays, such as for large-scale cohort studies. The MIRA R package can be accessed via Bioconductor, and comes with multiple vignettes demonstrating how to apply it to biological data. MIRA provides a novel tool to enhance analysis of DNA methylation and leverage existing data from regulatory assays to gain new regulatory insights.

## Funding

This work has been supported by the University of Virginia and by an NIH training grant to J.L. (NLM; 5T32LM012416).

*Conflict of Interest*: none declared.

## References

[bty083-B2] Farlik M. et al (2015) Single-cell DNA methylome sequencing and bioinformatic inference of epigenomic cell-state dynamics. Cell Rep., 10, 1386–1397. 25732828 10.1016/j.celrep.2015.02.001 PMC4542311

[bty083-B4] Kapourani C.-A., Sanguinetti G. (2016) Higher order methylation features for clustering and prediction in epigenomic studies. Bioinformatics, 32, i405–i412. 27587656 10.1093/bioinformatics/btw432

[bty083-B5] Sheffield N.C., Bock C. (2016) LOLA: enrichment analysis for genomic region sets and regulatory elements in R and Bioconductor. Bioinformatics, 32, 587–589. 26508757 10.1093/bioinformatics/btv612 PMC4743627

[bty083-B6] Sheffield N.C. et al (2017) DNA methylation heterogeneity defines a disease spectrum in ewing sarcoma. Nature Medicine, 23, 386–395. 10.1038/nm.4273 PMC5951283 28134926

[bty083-B7] Stadler M.B. et al (2011) DNA-binding factors shape the mouse methylome at distal regulatory regions. Nature, 480, 490–495. 22170606 10.1038/nature10716

[bty083-B9] Wijetunga N.A. et al (2017) A pre-neoplastic epigenetic field defect in HCV-infected liver at transcription factor binding sites and polycomb targets. Oncogene, 36, 2030–2044. 27721404 10.1038/onc.2016.340 PMC5383522

[bty083-B10] Yao L. et al (2015) Inferring regulatory element landscapes and transcription factor networks from cancer methylomes. Genome Biol., 16, 105. 10.1186/s13059-015-0668-3 PMC4460959 25994056

[bty083-B11] Zhu H. et al (2016) Transcription factors as readers and effectors of DNA methylation. Nat. Rev. Genet., 17, 551–565. 27479905 10.1038/nrg.2016.83 PMC5559737

